# The IGF2/IGF1R/Nanog Signaling Pathway Regulates the Proliferation of Acute Myeloid Leukemia Stem Cells

**DOI:** 10.3389/fphar.2018.00687

**Published:** 2018-06-29

**Authors:** Dan-dan Xu, Ying Wang, Peng-jun Zhou, Shu-rong Qin, Rong Zhang, Yi Zhang, Xue Xue, Jianping Wang, Xia Wang, Hong-ce Chen, Xiao Wang, Yu-wei Pan, Li Zhang, Hai-zhao Yan, Qiu-ying Liu, Zhong Liu, Su-hong Chen, Hong-yuan Chen, Yi-fei Wang

**Affiliations:** ^1^College of Life Science and Technology, Jinan University, Guangzhou, China; ^2^Guangdong Provincial Key Laboratory of Bioengineering Medicine, Jinan University, Guangzhou, China; ^3^Guangdong Food and Drug Vocational College, Guangzhou, China; ^4^State Key Laboratory of Oncology in South China and Collaborative Innovation Center for Cancer Medicine, Sun Yat-sen University Cancer Center, Guangzhou, China; ^5^Department of Biochemistry and Molecular Medicine, Cancer Center, George Washington University School of Medicine and Health Sciences, Washington, DC, United States; ^6^Department of Pathogen Biology and Immunology, School of Basic Course, Guangdong Pharmaceutical University, Guangzhou, China; ^7^College of Pharmacy, Jinan University, Guangzhou, China; ^8^Interdisciplinary Graduate School of Medicine and Engineering, University of Yamanashi, Kofu, Japan

**Keywords:** Nanog, IGF2, IGF1R, proliferation, leukemia stem cells

## Abstract

Acute myeloid leukemia is an aggressive disease characterized by clonal proliferation and differentiation into immature hematopoietic cells of dysfunctional myeloid precursors. Accumulating evidence shows that CD34^+^CD38^-^ leukemia stem cells (LSCs) are responsible for drug resistance, metastasis, and relapse of leukemia. In this study, we found that Nanog, a transcription factor in stem cells, is significantly overexpressed in CD34^+^ populations from patients with acute myeloid leukemia and in LSCs from leukemia cell lines. Our data demonstrate that the knockdown of Nanog inhibited proliferation and induced cell cycle arrest and cell apoptosis. Moreover, Nanog silencing suppressed the leukemogenesis of LSCs in mice. In addition, we found that these functions of Nanog were regulated by the insulin-like growth factor receptor (IGF1R) signaling pathway. Nanog overexpression rescued the colony formation ability of LSCs treated with picropodophyllin (PPP), an IGF1R inhibitor. By contrast, knockdown of Nanog abolished the effects of IGF2 on the colony formation ability of these LSCs. These findings suggest that the IGF2/IGF1R/Nanog signaling pathway plays a critical role in LSC proliferation.

## Introduction

Acute myeloid leukemia is a cancer of myeloid blood cells in the bone marrow (Fialkow et al., 1987). Despite progress made in the treatment of AML, most patients suffer relapses of the disease (Carella et al., 2013). In the past 40 years, chemotherapy regimens for AML generally included cytarabine in combination with anthracycline (Peloquin et al., 2013). Although these treatments can often eliminate part of the leukemia cells and extend the life span of the patients, the 5-year survival rate of young patients is still below 40% (Roboz and Guzman, 2009).

Leukemia stem cells were first discovered by Lapidot et al. (1994). According to previous studies, LSCs are considered to be responsible for drug resistance, metastasis, and tumor initiation and relapse (Sands et al., 2013) and have been described to have many properties that distinguish them from the general blast population (Felipe Rico et al., 2013). Therefore, it is important to target these malignant cells and investigate their intricate mechanisms to improve outcomes in patients. In recent years, the homeodomain-containing transcription factor Nanog has received increasing attention because of its pivotal roles in tissue development, stem cell maintenance, and tumor progression (Jeter et al., 2009; Zbinden et al., 2010; Noh et al., 2012). Nanog was discovered in 2003 in a screening for factors that maintain self-renewal of mouse embryonic stem cells (ESCs) in the absence of leukemia inhibitory factor (LIF) signaling pathways (Chambers et al., 2003; Mitsui et al., 2003). Together with Sox2 and Oct4, Nanog plays an important role in regulating self-renewal and maintaining the pluripotency of ESCs (Loh et al., 2006; Wang et al., 2006). Nanog is expressed in leukemia cells (Eberle et al., 2010), as well as in human solid tumors such as glioma, hepatocellular carcinoma, prostate cancer, pancreatic cancer, and leukemia (Eberle et al., 2010; Zbinden et al., 2010; Jeter et al., 2011; Shan et al., 2012; Lu et al., 2013). Moreover, accumulating evidence demonstrates that Nanog is essential for cancer cell proliferation, invasion, clonogenic growth, and tumorigenicity (Jeter et al., 2009, 2011; Zbinden et al., 2010; Ji and Jiang, 2013). A positive association between Nanog and tumor cell growth has also been reported (Jeter et al., 2015). Although it is not known whether this phenomenon is important to cell growth, increased proliferation is essential to tumor cells (Hanahan and Weinberg, 2011).

Ectopic expression of Nanog in human embryonic kidney HEK293 cells induces cell proliferation, anchor-independent growth in soft agar and, most importantly, tumor formation in athymic nude mice (Lin et al., 2011). By contrast, depletion of Nanog inhibits proliferation, reduces invasion, and is associated with increased apoptosis and S-phase arrest in human gastric cancer cells (Ji and Jiang, 2013). Other reports showed that Nanog regulates growth and proliferation of cancer stem cells (CSCs) in human hepatocellular carcinoma and glioma stem cells (Zbinden et al., 2010; Shan et al., 2012). In addition, Nanog promotes drug resistance, cell migration, and epithelial–mesenchymal transition (Chiou et al., 2010; Jeter et al., 2011). Depletion of both Nanog and Oct4 inhibits expression of the key epithelial–mesenchymal transition factor Slug and blocks tumorigenic and metastatic capacity in lung adenocarcinoma cells, as well as improves the mean survival of immunocompromised mice (Chiou et al., 2010). Moreover, Nanog knockdown reduces self-renewal, which is associated with decreased expression of stemness genes, that could be restored by overexpression of Nanog in Nanog-negative human hepatocellular carcinoma cells (Shan et al., 2012). Other studies have shown that double knockdown of Nanog and Oct4 significantly reduces proliferation, migration, invasion, chemoresistance, and tumorigenesis of pancreatic cancer cells *in vitro* and *in vivo* (Lu et al., 2013). In our previous study, we found that Nanog was overexpressed in LSCs from leukemia cell lines (Xu et al., 2016). However, it is not known whether Nanog is active in LSCs and whether its function in these cells is similar to that in solid tumor cells.

Insulin-like growth factor 1 receptor (IGF-1R), a receptor tyrosine kinase, is activated by binding of its ligands IGF1 and IGF2 (Yuen and Macaulay, 2008). Evidence suggests that IGF1R and its ligands are involved in the development and progression of cancer (Baserga et al., 1997). IGF1R activation or overexpression mediates several aspects of the malignant phenotype (Hakam et al., 1999; Osuka et al., 2013). More importantly, high levels of IGF1R expression are required for leukemia-initiating cell activity in T-cell acute lymphoblastic leukemia, and inhibition of IGF1R blocks the growth and viability of T-cell acute lymphoblastic leukemia cells (Medyouf et al., 2011). Recruitment of these molecules activates signaling via the phosphatidylinositol-3-kinase (PI3K)-AKT (Baserga et al., 2003; Manning and Cantley, 2007). In many studies phosphorylation of IGF1R was inhibited, which reduced Akt activation, enhanced cancer cell apoptosis, and suppressed tumor cell growth (Chakravarti et al., 2002; Carboni et al., 2009; Hou et al., 2011).

Therefore, it is important to understand the correlation between Nanog and its regulators. In our previous study, we studied the correlation between Nanog and microRNAs (miR-150) (Xu et al., 2016). Importantly, in other previous studies, we found that IGF2 was overexpressed in CD34^+^CD38^-^ LSCs (Zhang et al., 2015). Moreover, Chen et al. (2014) demonstrated that IGF1R signaling activation in cancer cells in the presence of cancer-associated fibroblasts expressing IGF2 can induce Nanog expression and promote stemness, and that IGF2 secreted by cancer cells instigates fibroblasts and bone marrow-derived vascular progenitor cells to promote cancer progression (Xu W. et al., 2017).

Although IGF2 and Nanog have been known to play an important part in regulating proliferation of cancer cells, it remains unclear as to how they cooperate in regulating proliferation of LSCs in AML. Here, we report that Nanog is significantly overexpressed in CD34^+^ cell populations from patients with AML and in LSCs from leukemia cell lines. More importantly, our data suggest that IGF2/IGF1R/Nanog signaling axis plays a key role in the proliferation of LSCs.

## Materials and Methods

### Primary Cell Isolation

Leukemia stem cells from human leukemia cell lines KG-1a and MOLM13 were isolated and identified according to the cell markers CD34^+^CD38^-^, as we previously described (Zhang et al., 2015, 2016; Xu et al., 2016), and cultured in RPMI 1640 medium supplemented with 10% fetal bovine serum and 1% penicillin and streptomycin at 37°C under a humidified atmosphere of 5% CO_2_. LSCs from KG-1a and MOLM13 were isolated using a magnetic-activated cell-sorting (MACS) kit (Cat No. 130-056-701; Miltenyi Biotec, Bergisch Gladbach, Germany) and cultured in serum-free IMDM (STEMCELL Technologies, Vancouver, BC, Canada) containing 20 ng/mL basic fibroblast growth factor (bFGF; PeproTech, Rocky Hill, NJ, United States), 20 ng/mL epidermal growth factor (EGF; PeproTech), and B27 media (1:50; Life Technologies, Carlsbad, CA, United States).

Human peripheral blood samples were collected from The First Affiliated Hospital of Jinan University. The blood of patients with AML was sampled, and all the participants provided informed consent. The study protocol was approved by the ethics committee of The First Affiliated Hospital of Jinan University. Mononuclear cells were obtained using density gradient centrifugation, and CD34^+^ leukemia cells were enriched by magnetic microbeads (Miltenyi Biotec). The CD34^+^ leukemia cells were cultured in hematopoietic stem cell medium (Stem Cell Technologies, Vancouver, BC, Canada) supplemented with B27 (Gibco, Grand Island, NY, United States), 20 ng/mL EGF, and 20 ng/mL bFGF.

### Reagents and Lentiviral Infections

The IGF1R inhibitor picropodophyllin (PPP) and 5-bromo-2′-deoxyuridine (BrdU) were purchased from Sigma-Aldrich (St. Louis, MO, United States). Nanog-targeting shRNA lentiviral constructs were purchased from GeneChem (Shanghai, China). The sequences of these shRNAs are 5′-GGGTTAAGCTGTAACATACTT-3′ for Nanog1 shRNA, targeting the 3′-UTR, and 5′-GCATGCAGTTCCAGCCAAATT-3′ for Nanog2 shRNA, targeting the coding sequence (Zaehres et al., 2005; Zbinden et al., 2010; Shan et al., 2012). Nanog vector (pcDNA3.1-Nanog) was purchased from GenePharma (Shanghai, China) (Xu et al., 2016). LSCs were transduced with lentiviral constructs in a medium containing 5 μg/mL polybrene, according to the manufacturer’s protocol.

### Proliferation and Apoptosis Assays

Leukemia stem cell proliferation analysis was assessed by using trypan blue (Beyotime, Haimen, China). LSCs (1 × 10^4^) were seeded into a 48-well plate containing 200 μL of serum-free IMDM media supplemented with 20 ng/mL bFGF, 20 ng/mL EGF, and B27 media (1:50). Subsequently, the 48-well plate was incubated for 4 h at 37°C in 5% CO_2_ in a humidified incubator. After 72 h, the LSCs were counted by trypan blue staining. For detection of the cell proliferation marker Ki-67, 1 × 10^5^ LSCs were incubated with an antibody against Ki-67 (Cell Signaling Technology) and washed three times with Tris-buffered containing 0.1% Tween-20. Subsequently, the cells were incubated with an FITC-conjugated goat secondary antibody for 0.5 h at 37°C. Nuclei were counterstained with DAPI. The cells were photographed by fluorescence microscope (Carl Zeiss, Thornwood, NY, United States) (Xu et al., 2016). For apoptosis assessment, LSCs were transfected with shRNAs and cultured for 72 h. The cells were collected, washed, and 5 μL of binding reagent and 5 μL of annexin V-APC (KeyGen Biotech, Nanjing, China) were added. After 30 min, cells were washed thrice with PBS and stained with 5 μL of 7-AAD for 20 min, according to the manufacturer’s instructions. The experiments were repeated thrice. All data were analyzed by using the FlowJo software (San Diego, CA, United States).

### Sphere Formation and Soft Agar Assays

For sphere formation, according to our previous studies (Xu et al., 2016; Zhang et al., 2016), for sphere formation assay, 500 LSCs were cultured in an ultralow attachment 6-well plate with IMDM medium (STEMCELL Technologies, Vancouver, BC, Canada) with recombinant human EGF (20 ng/ml; Pepro Tech), recombinant human bFGF (20 ng/ml, Pepro Tech), B27 supplement (1:50, Invitrogen, Carlsbad, CA, United States) without different concentration of IGF2 and PPP. Culture media was replenished every 3 days about 2 weeks later, the number of LSCs formation was counted and the colonies efficiency (more than 50 cells) was calculated. For soft agar assay, a 6-well plate was coated with bottom agar layer containing IMDM supplemented with recombinant human EGF (20 ng/mL), recombinant human bFGF (20 ng/mL), B27 supplement (1:50), and different concentration of IGF2 or without IGF2. Top agar contained a single cell suspension of 500 LSCs in IMDM supplemented with recombinant human EGF (20 ng/mL), recombinant human bFGF (20 ng/mL), B27 supplement (1:50), and different concentrations of IGF2 or without IGF2. After 25 days, colonies of more than 50 cells were visualized as positive and stained with 0.5% crystal violet for 30 min at 37°C (Song et al., 2014).

### Quantitative Polymerase Chain Reaction (qPCR)

Total RNA was extracted from LSCs with Trizol Reagent (Tiangen, Beijing, China), treated with RNAse-free DNAse (Takara Biotechnology, Dalian, China), and reverse-transcribed into cDNA using PrimerScript Master mix (Takara Biotechnology) according to the manufacturer’s instructions. qPCR was performed on an RT-PCR system (CFX96 Real-Time System; Bio-Rad, Shanghai, China) using appropriate primers (Sangon Biotech, Shanghai, China). In clinical samples, Nanog and IGF1R mRNA expression levels were calculated relative to those in normal blood. The following PCR conditions were used on a Light Cycler: 95°C for 5 s, 60°C for 5 s, followed by 42 cycles at 95°C for 15 s, and 60°C for 1 min in a 20-μL reaction volume. Gene-specific primers are shown in Supplementary Table S1. Relative expression level was calculated using the 2^-(ΔΔ^*^C^*^t)^ method with GAPDH as the reference gene. All the experiments were replicated thrice. Student’s *t*-test was used to evaluate the differences between mRNA expression levels of the two groups. SPSS 19.0 (SPSS Inc., Chicago, IL, United States) was employed to calculate the difference.

### Western Blot Analysis

Treated LSCs were lysed with RIPA (Tiangen), proteins were subjected to SDS-PAGE, and transferred to a PVDF membrane (Immobilon-P^SQ^ transfer membrane, Millipore, NY, United States). The membrane was washed in blocking buffer [5% skimmed milk (Gibco) containing 0.1% Tween-20] and incubated with antibodies overnight. The dilution of the primary antibodies against Nanog (#3580, Cell Signaling Technology; #ab190250, Abcam; #AF1997, R&D Systems) was 1:1000. Antibodies against IGF1R, p-IG1R, Akt, and p-Akt were purchased from Cell Signaling Technology, and the dilutions were 1:1000 according to the manufacturer’s instructions. The following day, the membrane was washed by TBST buffer (0.1% Tween-20 in Tris-base) thrice. The membrane was incubated with horseradish peroxidase-conjugated secondary antibodies (1:5000). The bound proteins were detected using chemiluminescence detection kit ECL (Millipore). GAPDH was used as an internal control. The images were acquired on a Canon scanner (Canon, Tokyo, Japan) and processed by Adobe Photoshop CS5 (San Jose, CA, United States). The relative density of bands was determined by using the Image J software (NIH, Bethesda, MD, United States).

### BrdU Incorporation Assay

Cells were fixed in 4% paraformaldehyde at room temperature for 20 min and washed thrice. Next, the cells were permeabilized with 0.1% Triton X-100 for 30 min and washed thrice. The cells were incubated with mouse anti-BrdU primary antibody at room temperature for 1.5 h after blocking with 10% anti-goat serum in PBS. Then, cells were incubated with secondary antibody conjugated with Alexa Flour 488 and visualized using confocal microscopy (Carl Zeiss, Thornwood, NY, United States).

### Animal Experiments

NOD/SCID and BALB/c mice were housed and bred under specific pathogen-free conditions. All animal procedures were approved by and conducted according to the Jinan University Laboratory Animal Ethics Committee.

In the xenograft model, mice were divided into two groups. Female NOD/SCID mice (5-week-old, *n* = 6) were intravenously injected via the tail vein with 2 × 10^5^ LSCs in which Nanog was depleted, using Nanog1 shRNA. In the negative control group, mice (*n* = 6) were intravenously injected via the tail vein with 2 × 10^5^ LSCs transduced with a control shRNA (shGFPctrl) lentiviral vector. Mice were euthanized after 60 days, and peripheral blood, bone marrow, and spleens were collected. Human leukemia cells from mouse tissues were evaluated by flow cytometry. Human leukemia cells were identified as CD45^+^ cells (hCD45^+^) (Ferretti et al., 2012).

In the subcutaneous model, female BALB/c mice (5-week-old, *n* = 6) were inoculated subcutaneously in the flank with 2 × 10^5^ LSCs in which Nanog was silenced using Nanog1 shRNA. In the negative control group, mice (*n* = 6) were injected with 2 × 10^5^ LSCs transduced with shGFPctrl lentiviral vector. After 4 weeks, the mice were euthanized, and the weight of tumors harvested from the mice was measured.

### Statistical Analysis

Data are presented as the mean ± standard deviation (SD). Student’s *t*-test was used to compare differences in cell proliferation, apoptosis, cell cycle arrest, sphere size, and mRNA expression levels between the groups. Correlations between the levels of Nanog and IGF1R were assessed using Spearman’s rank correlation analysis. All analyses were performed using SPSS version 19 (SPSS Inc., Chicago, IL, United States). *P-*value < 0.05 was considered statistically significant, ^∗^*P* < 0.05 and ^∗∗^*P* < 0.01.

## Results

### Nanog Is Highly Expressed in Leukemia Stem Cell Populations and Promotes Leukemia Stem Cell Proliferation

**FIGURE 1:**
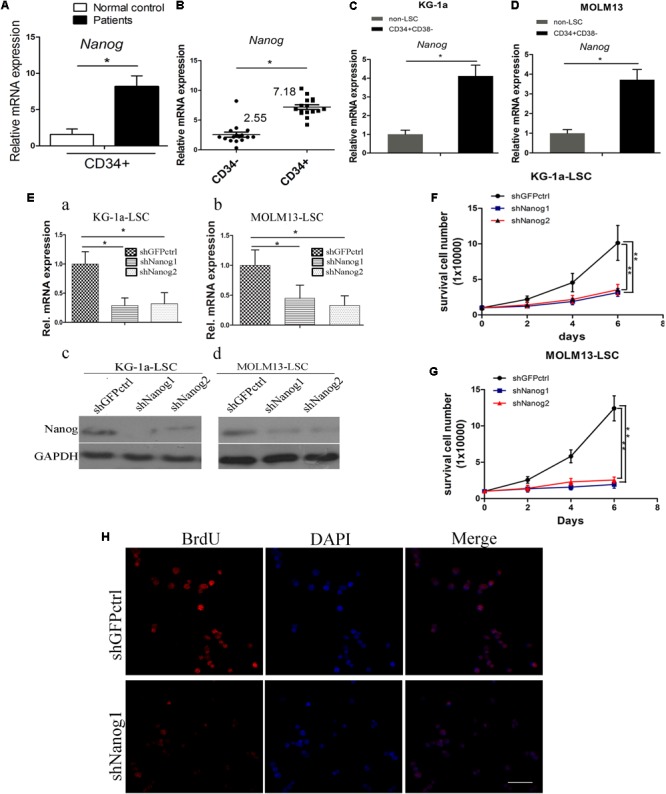
Nanog is highly expressed in leukemia stem cell (LSC) populations, and promotes LSC proliferation. **(A)** qPCR analysis of Nano*g* mRNA level in a panel of 16 samples of leukemia cells derived from patients with AML. **(B)** Nanog mRNA level in CD34^+^ cells of patients with AML compared with the counterpart CD34^-^ cells. **(C,D)** qPCR analysis of Nanog mRNA level in LSCs from KG-1a and MOLM13 cell lines. **(E)** qPCR **(a,b)** and western blot **(c,d)** analysis of the knockdown efficiency of shNanog1 and shNanog2. **(F,G)** Proliferation curve analysis of LSCs after silencing of Nanog. Cells (1 × 10^4^) were stably transduced with lentiviral constructs carrying shGFPctrl, shNanog1, or shNanog2. After 72 h, proliferation of LSCs was analyzed by trypan blue staining. The experiments were repeated three times independently (^∗^*P* < 0.05). **(H)** BrdU analysis of LSC proliferation after knockdown of Nanog. Immunofluorescence expression analysis of Nanog using a rabbit monoclonal antibody after silencing of Nanog. Grayscale analysis was done by using ImagePro Plus (^∗^*P* < 0.05). Scale bar, 100 μm.

The expression level of Nanog in LSCs was analyzed in CD34^+^ cells isolated from blood samples of patients with AML (Supplementary Table S2) using MACS according to our previous study (Zhang et al., 2015) and in CD34^+^CD38^-^ LSCs isolated from AML cell lines KG-1a and MOLM13. There was a significant increased expression of Nanog in patients CD34^+^ cells, as compared with in normal control (**Figure 1A**). qPCR analysis demonstrated that Nanog mRNA levels were significantly higher in CD34^+^ leukemia cells than in CD34^-^ counterparts (**Figure 1B**), and overexpressed in LSCs (CD34^+^CD38^-^) (**Figures 1C,D**). In addition, the mRNA levels of Sox2 and Bmi1 were higher in KG-1a LSCs than in non-LSCs (CD34^+^CD38^+^, CD34^-^CD38^+^, CD34^-^CD38^-^) (Supplementary Figure S1).

To explore the biological role of Nanog in LSCs, we performed shRNA experiments to target Nanog mRNA. The efficiencies of shRNAs against Nanog are shown in **Figure 1E**. shNanog1 and shNanog2 markedly resulted in a decrease in Nanog of approximately 60–70% at the RNA level and protein level. We found that Nanog shRNAs significantly reduced cell proliferation of KG-1a LSCs and MOLM13 LSCs compared with a control shGFPctrl (**Figures 1F,G**). We further investigated the spheroid formation capacity of LSCs cultured in IMDM supplemented with 20 ng/mL EFG, 20 ng/mL bFGF, 100 ng/mL IGF2, and B27 in soft agar plate experiments. The results showed that both shNanog1 and shNanog2 significantly decreased colony formation efficiencies of KG-1a LSCs and MOLM13 LSCs (Supplementary Figure S2). Consistently, BrdU incorporation analysis demonstrated that silencing of Nanog resulted in a 30–40% reduction in cell proliferation (**Figure 1H**). Altogether, these results demonstrate that Nanog plays an important role in regulating LSC proliferation.

### Nanog Knockdown Induces Cell Cycle Arrest and Apoptosis of Leukemia Stem Cells

**FIGURE 2:**
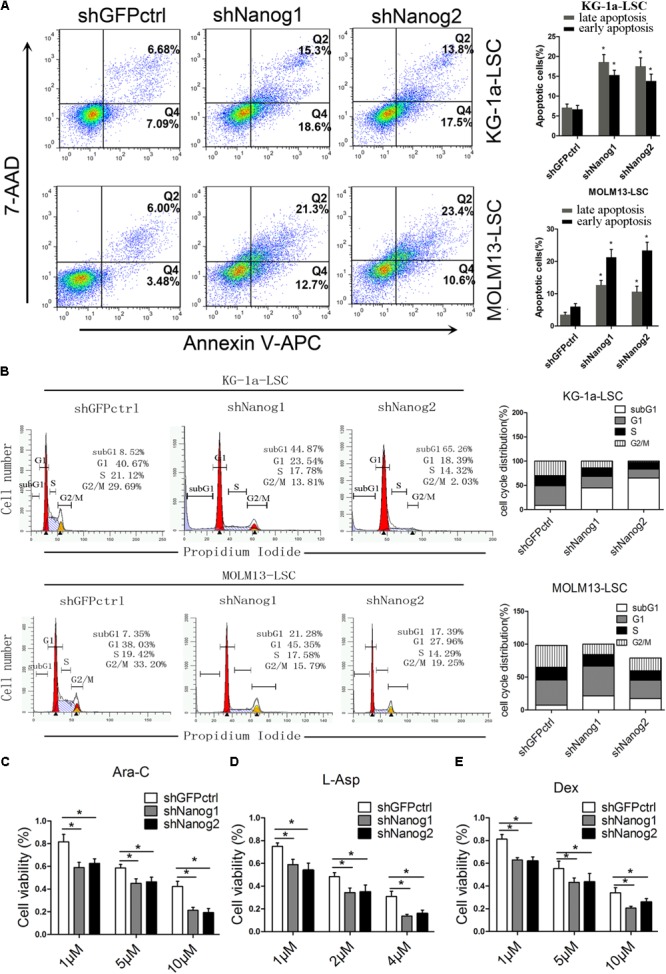
Nanog knockdown induces cell cycle arrest and apoptosis of LSCs. **(A)** Flow cytometry analysis of apoptosis after Nanog knockdown. LSCs were transduced with shNanog1, shNanog2, or shGFPctrl. Following 48 h, apoptosis was evaluated by flow cytometry. The experiments were repeated independently three times (^∗^*P* < 0.05). Data are presented as the mean ± SD. **(B)** Flow cytometry analysis of subG1 percentages in LSCs of KG-1a and MOLM13 cell lines was conducted by using the FlowJo software. The experiments were repeated independently in triplicate (^∗^*P* < 0.05). **(C–E)** Cell viability after knockdown of Nanog. In total, 5 × 10^3^ cells transduced with lentiviral vector were seeded in IMDM containing Ara-C, L-Asp, or dexamethasone. After culture for 48 h, CCK-8 was added to the cell culture and incubated for 4 h. Absorbance was recorded at 450 nm by using a microplate absorbance reader (*n* = 3).

To further investigate the function of Nanog on LSCs proliferation, we conducted flow cytometric analysis to compare the cell cycle profiles of LSCs treated with Nanog shRNA. 7-AAD/annexin V-APC analysis demonstrated that knockdown of Nanog significantly increased the number of apoptotic cells at both the early and late stage of apoptosis (**Figure 2A**). The percentage of cells in subG1 phase was significantly increased upon treatment of Nanog knockdown in both KG-1a LSCs and MOLM13 LSCs (**Figure 2B**). The subG1 percentages in shNanog1 and shNanog2-treated versus shGFPctrl-treated KG-1a LSCs were 44.87, 65.26, 8.52%, respectively (**Figure 2B**). Also, The subG1 percentages in shNanog1 and shNanog2-treated versus shGFPctrl-treated MOLM13 LSCs were 21.28, 17.39, 7.35%, respectively (**Figure 2B**). To further investigate the cell viability of LSCs after Nanog silencing, the three drugs Ara-C, L-Asp, and dexamethasone were used for leukemia treatment. As shown in **Figure 2C**, LSCs were more sensitive to Ara-C after Nanog silencing. We also found similar results with the other two drugs (**Figures 2D,E**). Taken together, these results demonstrated that knockdown of Nanog induces cell apoptosis of LSCs and cell cycle arrest at the G0/G1 phase and decreases the drug resistance of LSCs.

### Nanog Depletion Suppresses Stemness Factor Levels and Colony Formation

**FIGURE 3:**
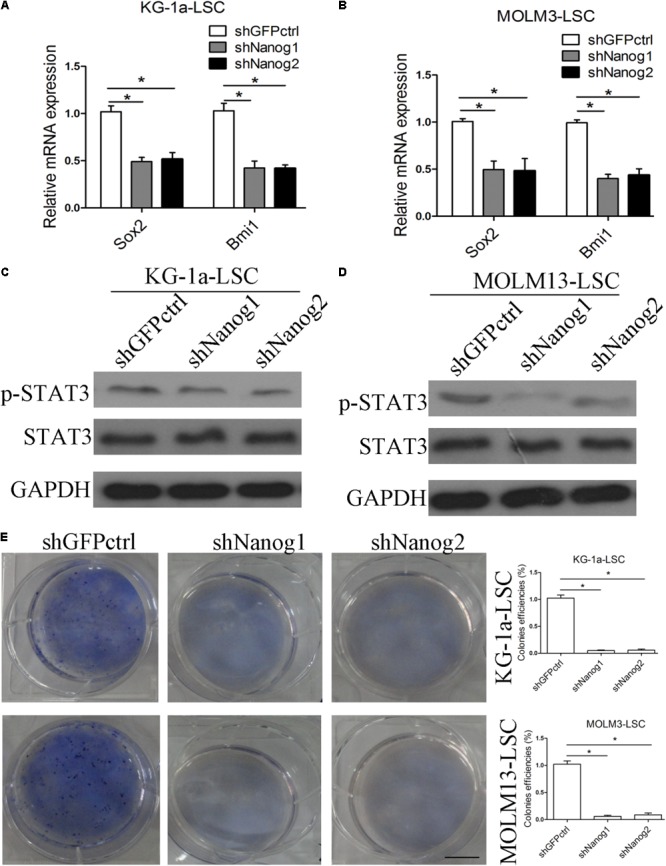
Silencing of Nanog suppresses stemness factor levels and proliferation of LSCs. **(A,B)** qPCR data demonstrate that the levels of Sox2 and Bmi1 was decreased. LSCs were transduced with lentiviral vector. After 72 h, LSCs were harvested. The experiments were repeated independently three times (^∗^*P* < 0.05). Data are presented as the mean ± SD. **(C,D)** Western blot analysis shows that p-STAT3 level was reduced. LSCs were transduced with lentiviral vectors. After 72 h, LSCs were harvested and analyzed. **(E)** Soft agar plates experiments demonstrate that Nanog knockdown inhibited the proliferation of LSCs. LSCs (500) were seeded in IMDM with IGF2 (20 ng/mL), EGF (20 ng/mL), bFGF (20 ng/mL), B27 supplement (1:50). After 2 weeks later, the colonies of LSCs were large enough to be visualized, and were stained with 0.5% crystal violet for 30 min at 37°C.

Chiou et al. (2008) showed positive correlations between Nanog and Oct4 in oral cancer stem-like cells and in high-grade oral squamous cell carcinoma. In addition, co-expression of Oct4 and Nanog enhances malignancy in lung adenocarcinoma by inducing CSC-like properties and epithelial–mesenchymal transition (Chiou et al., 2010). Therefore, we questioned the relation between Nanog and other stemness factors. We thus performed qPCR experiments to analyze the expression level of stemness factors. As expected, the expression levels of Sox2 and Bmi1 were decreased in both LSCs (**Figures 3A,B**). We also examined p-STAT3 expression level and found that in both LSCs, Nanog depletion reduced *p*-STAT3 level (**Figures 3C,D**). Moreover, Nanog knockdown suppressed the growth and proliferation of LSCs, as determined by a colony formation assay (**Figure 3E**).

### Nanog Knockdown Impairs Leukemogenesis of Leukemia Stem Cells *in Vivo*

**FIGURE 4:**
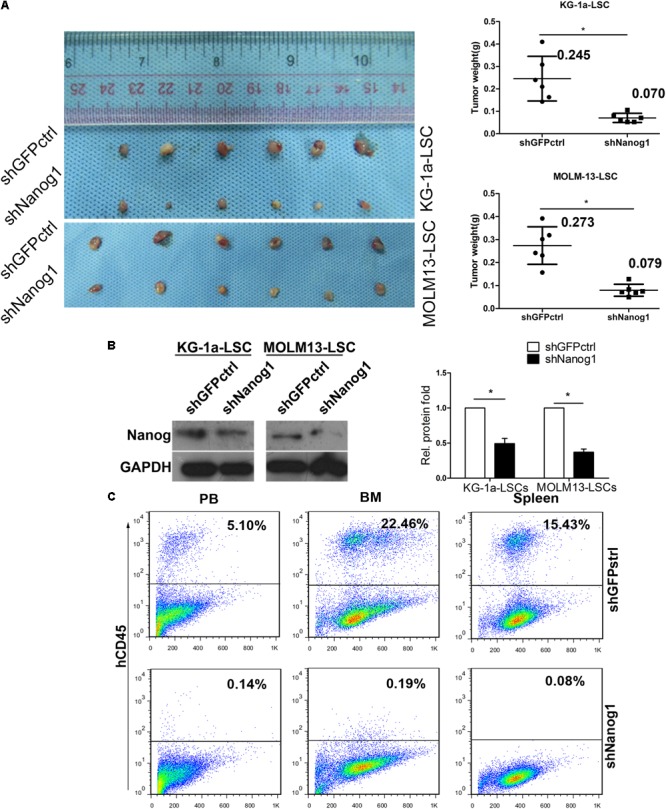
Knockdown of Nanog impairs LSC growth *in vivo*. **(A)** BALB/c nude mice were injected subcutaneously with KG-1a LSCs or MOLM13 LSCs transduced with shGFPctrl or shNanog1 and shNanog2. In total, 2 × 10^5^ treated cells were injected subcutaneously into BALB/c nude mice. Tumors were harvested at 30 days after injection. Data are presented as the mean ± SD (^∗^*P* < 0.05). **(B)** Western blot analysis of Nanog protein level excised from tumors. **(C)** Representative experiment data of hCD45 expression in PB, BM, and spleen harvested from NOD/SCID mice injected with shGFPctrl (Top) or shNanog1 (Bottom) KG-1a LSCs. NOD/SCID mice were injected via the tail vein with 2 × 10^5^ KG-1a LSCs. Mice were euthanized 60 days after injection. PB, BM, and spleen were collected and subjected to analysis. PB, peripheral blood; BM, bone marrow.

To investigate the biological role and function of Nanog *in vivo*, we first established xenograft tumor models with KG-1a LSCs and MOLM13 LSCs and evaluated the effect of Nanog knockdown on tumor growth. Knockdown of Nanog remarkably suppressed the growth of KG-1a LSC xenograft tumors, as well as MOLM13 LSC xenograft tumors, in BALB/c nude mice (**Figure 4A**). The mean weight of excised tumors in Nanog-knockdown xenograft was threefold smaller than that in control tumors (**Figure 4A**). Furthermore, western blot analysis confirmed Nanog downregulation in Nanog-knockdown xenograft tumors (**Figure 4B**).

To further confirm the tumorigenic function of Nanog in LSCs, immunodeficient NOD/SCID mice were injected with KG-1a LSCs transduced with shGFPctrl or shNanog1 via the tail vein. Representative images of hCD45^+^ staining in peripheral blood, bone marrow, and spleen obtained from NOD/SCID mice are shown in **Figure 4C**. As expected, the proportion of hCD45^+^ cells was 17-fold lower in peripheral blood from mice injected with KG-1a LSCs treated with shNanog1 than in that treated with shGFPctrl (**Figure 4C**). Similar results were obtained in bone marrow and spleen (**Figure 4C**). These results suggest that Nanog plays an important role in regulating the leukemogenesis function of LSCs.

### The IGF2 Signaling Pathway Is Essential in the Regulation of Nanog Expression in Leukemia Stem Cells

**FIGURE 5:**
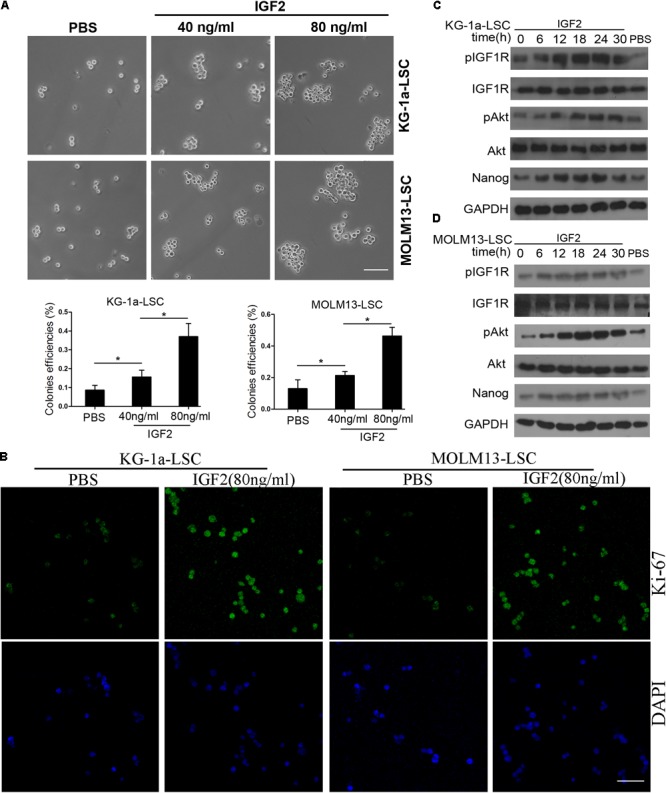
Insulin-like growth factor (IGF) signaling regulates Nanog expression and LSC growth. **(A)** IGF2 significantly increased the colony formation efficiencies of KG-1a LSCs and MOLM13 LSCs. LSCs were cultured in IMDM containing bFGF (20 ng/mL), EGF (20 ng/mL), and B27. All data represent three independent experiments for each condition (^∗^*P* < 0.05). Scale bar, 100 μm. **(B)** Treatment with IGF2 promoted LSC proliferation. LSCs were cultured in IMDM containing bFGF (20 ng/mL), EGF (20 ng/mL), and B27 with or without IGF2 (100 ng/mL) for 72 h. The cells were then harvested and incubated with an antibody against Ki-67. Scale bar, 60 μm. **(C,D)** Western blot analysis showed that IGF2 increased p-IGF1R, p-Akt, and Nanog expression levels in a time-dependent manner. GAPDH was used as a loading control. The experiments were independently repeated three times.

In our previous study, we found that IGF2 is highly expressed in CD34^+^CD38^-^ KG-1a cells (Zhang et al., 2015). Therefore, we investigated whether IGF2 signaling influences Nanog-mediated LSC proliferation. Sphere formation assays showed that similar to Nanog knockdown, treatment with IGF2 significantly increased the colony formation efficiencies of LSCs (**Figure 5A**). The immunofluorescence results showed that IGF2 significantly promoted the proliferation of LSCs according to the expression level of Ki-67 (**Figure 5B**). Furthermore, we found that treatment with IGF2 increased the phosphorylation levels of IGF1R and its downstream protein Akt in a time-dependent manner (**Figures 5C,D**). More importantly, treatment with IGF2 significantly elevated Nanog expression both in KG-1a LSCs and MOLM13 LSCs (**Figures 5C,D**). In addition, Nanog knockdown abrogated the pro-proliferative effect of IGF2 (Supplementary Figure S3). These results suggest that IGF2/IGF1R signaling is activated upstream of Nanog.

### Inhibition of IGF1R Suppresses the Expression Level of IGF2-Induced Nanog

**FIGURE 6:**
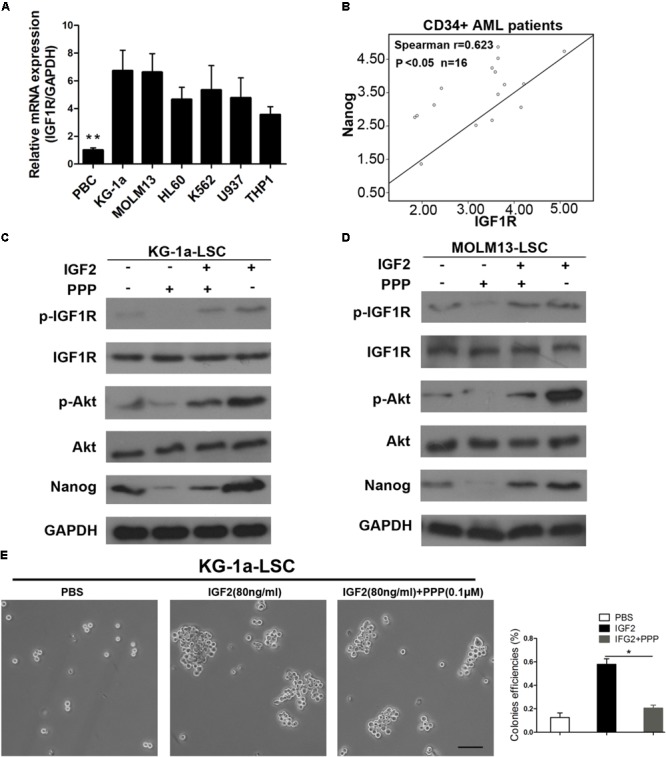
Inhibition of IGF1R suppresses LSC proliferation. **(A)** qPCR analysis of IGF1R expression in healthy PB cells and leukemia cell lines. Data are presented as the mean ± SD (^∗∗^*P* < 0.01). **(B)** Correlation between Nanog and IGF1R mRNA expression levels as determined by qPCR and Spearman’s analysis (^∗^*P* < 0.05). Normal blood was used as internal control. **(C,D)** Western blot analysis of p-IGF1R, p-Akt, and Nanog expression after IGF1R inhibition by PPP (0.1 μM) in both LSC lines. **(E)** Colony formation detection of LSCs upon addition of IGF2 and the IGF1R inhibitor PPP. Scale bar, 100 μm. PB, peripheral blood; PPP, picropodophyllin.

Insulin-like growth factor receptor activation or overexpression mediates several aspects of the malignant phenotype (Hakam et al., 1999; Osuka et al., 2013). Thus, we tested IGF1R mRNA levels in several leukemia cell lines. We found that IGF1R mRNA level was significantly higher in leukemia cells than in normal blood (**Figure 6A**). Next, we evaluated IGF1R and Nanog expression levels in blood samples from patients with AML. As shown in **Figure 5B**, IGF1R mRNA expression showed a highly significant (*P* < 0.0001) positive correlation with Nanog mRNA expression in CD34^+^ leukemia cells (**Figure 6B**). Furthermore, we found that IGF1R inhibitor PPP reversed IGF2-induced Nanog expression (**Figures 6C,D**), as well as IGF2-induced IGF1R and Akt phosphorylation (**Figures 6C,D**). In addition, we examined whether IGF1R was important in LSC proliferation. As expected, the results of sphere formation experiments demonstrate that PPP significantly decreased colony formation efficiencies (**Figure 6E**). Moreover, PPP inhibited LSC proliferation, which was rescued partly by Nanog overexpression (Supplementary Figure S4). Taken together, these results suggest that extracellular IGF2 signaling is responsible for Nanog expression in LSCs.

## Discussion

Acute myeloid leukemia is a stem cell-related disease consisting of hematopoietic neoplasms, with a decreased capacity to differentiate into normal and mature cells (Peloquin et al., 2013). Laboratory research data suggest that AML originates from a subset of a rare population of cells that are capable of self-renewal and differentiation into malignant blasts (Roboz and Guzman, 2009).

Here, we describe a critical role for Nanog in the regulation of LSC proliferation. Our results demonstrate that Nanog is overexpressed in patient-derived CD34^+^ leukemia cells and in CD34^+^CD38^-^ LSCs from leukemia cell lines. We show that depletion of Nanog decreases the proliferation of LSCs *in vitro* and progressively abolishes leukemogenesis *in vivo*. Our research strongly suggests that Nanog is important for LSC proliferation. Moreover, we found evidence that Nanog regulates the proliferation of LSCs, which is mediated directly by the IGF2 signaling axis. Nanog is overexpressed in cancer cells (Jeter et al., 2009, 2011; Pan et al., 2010; Zbinden et al., 2010; Du et al., 2012, 2013; Shan et al., 2012; Zhang et al., 2013; Wang et al., 2017) and in this study, we found that Nanog is overexpressed in CD34^+^ cells of patients with AML and in CD34^+^CD38^-^ cells of leukemia cell lines. In clinical samples, the expression level of Nanog was higher in CD34^+^ cells than in CD34^-^ cells. However, the level of Nanog in normal blood cells remains unclear. Hence, Nanog as a potential therapeutic target in AML needs to be studied further.

Several studies have shown that Nanog regulates self-renewal of cancer cells and CSCs. Nanog regulates self-renewal of prostate, breast, and colon cancer cells, and knockdown of Nanog inhibits tumor development *in vivo* (Jeter et al., 2009). Additionally, Nanog regulates self-renewal of human hepatocellular carcinoma and is essential in regulating the growth of glioma stem cells (Zbinden et al., 2010; Shan et al., 2012). Consistent with these reports, our results show that knockdown of Nanog inhibits growth, and induces apoptosis and G0/G1 cell cycle arrest in LSCs. We demonstrate that silencing of Nanog reduced the growth properties of LSCs, including colony size.

However, the molecular mechanisms underlying Nanog-mediated proliferation of LSCs remain largely unknown. Understanding these mechanisms is essential to develop new strategies to target these cells. In this investigation, silencing of Nanog inhibited the proliferation of LSCs. This may be a transcriptional reprogramming effect mediated by Nanog. This hypothesis is consistent with the finding that Nanog is required for maintenance of self-renewal and pluripotency in ESCs (Chambers et al., 2003; Mitsui et al., 2003; Boyer et al., 2005; Loh et al., 2006). Nanog overexpression accelerates reprogramming in ESCs (Hanna et al., 2009), and the protein is critical for hindering differentiation of pluripotent cells. In addition, we found that Nanog expression level was positively correlated with the protein expression of IGF1R.

Insulin-like growth factor receptor is a receptor tyrosine kinase that is activated by binding to its ligands IGF1 and IGF2 (Yuen and Macaulay, 2008). Evidence suggests that IGF1R and its ligands are involved in the development and progression of cancer (Baserga et al., 1997). According to recent reports, IGF2 abnormalities have been demonstrated in many adult malignancies, in which its overexpression is positively correlated with poor prognosis (Singer et al., 2004; Lu et al., 2006). IGF2 secreted by cancer cells promotes cancer progression (Xu W. et al., 2017), and cancer-associated fibroblasts and IGF2 regulate the plasticity of lung cancer stemness via paracrine signaling (Chen et al., 2014). We previously found that IGF2 is overexpressed in LSCs (Zhang et al., 2015). However, the relationship between IGF2 and Nanog in LSCs is still unclear. Xu L. et al. (2017) demonstrated that IGF1/IGF1R/STAT3 signaling promotes gastric cancer growth and metastasis, but the mechanism was not elucidated. The exact molecular mechanism underlying IGF2 signaling and Nanog remains unclear. Here we also investigated whether stemness factor level including Bmi1, Sox2, and STAT3 were decreased after Nanog was knockdown. STAT3, an important transcription factor, is seen a stemness facor. STAT3 is important to both ESCs and CSCs. In many other studies other stemness facors Nanog, Oct4, and Sox2 play a key role in regualting ESCs and CSCs (Torres and Watt, 2008; Jeter et al., 2009; Zbinden et al., 2010).

In our study, IGF1R was essential for the proliferation of LSCs. Inhibition of IGF1R by PPP blocked Nanog expression and attenuated the colony formation capacity of LSCs. IGF1R is critical for tumorigenesis (Keku et al., 2012), particularly for the development and progression of tumorigenesis (Liu et al., 2014). The IGF1R signaling pathway is implicated in the pathogenesis and progression of cancer (Jenkins et al., 2012). High level of IGF1R expression is required for leukemia-initiating cell activity in T-ALL, and inhibition of IGF1R blocks the growth and viability of T-ALL cells (Medyouf et al., 2011). Additionally, IGF1R plays an important role in tumor cell metastasis and survival of malignant tumor cells (Sachdev et al., 2010), and is essential for the regulation of adaptive radioprotection in glioma stem cells (Osuka et al., 2013). Therefore, promising therapeutic agents targeting the IGF1R pathway include IGF1R monoclonal antibodies, IGF1R tyrosine kinase inhibitors, and IGF ligand-specific antibodies, which show good efficacy (Liu et al., 2014).

## Conclusion

Our results demonstrate that Nanog plays a key role in regulating the proliferation of LSCs. Silencing of Nanog decreased LSC proliferation, inducing apoptosis and cell cycle arrest and attenuating leukemogenesis of LSCs. Furthermore, we found that the Nanog mRNA expression-level is higher in LSCs from leukemia cell lines and CD34^+^ cells from AML clinical samples. IGF2/Nanog signaling axis regulates LSC proliferation and leukemogenesis of LSCs through Nanog. Altogether, our results show that the IGF2/IGF1R signaling axis is required for Nanog-regulated maintenance of LSC proliferation and indicate that Nanog is a downstream mediator of the IGF1R signaling pathway. These results provide new insights into the underlying mechanism of the IGF2/IGF1R/Nanog pathway in the proliferation of LSCs.

## Author Contributions

D-dX carried out most of the studies and wrote the manuscript. YW, P-jZ, and S-hC analyzed the data and results. RZ, YZ, XX, and S-rQ read and revised the entire manuscript. JW, XiaW, and H-cC provided technical assistance with several protocols. XW and Y-wP participated in the study design and wrote the paper. LZ and H-zY conceived the study. Q-yL, H-yC, and ZL participated in the design and coordination of the study. Y-fW designed the study and revised the manuscript. All authors have read and approved the final manuscript.

## Conflict of Interest Statement

The authors declare that the research was conducted in the absence of any commercial or financial relationships that could be construed as a potential conflict of interest.

## Abbreviations

AML: acute myeloid leukemia
IGF1R: insulin-like growth factor receptor
LSCs: leukemia stem cells
