# Supplementary material for: The IGF2/IGF1R/Nanog Signaling Pathway Regulates the Proliferation of Acute Myeloid Leukemia Stem Cells
Source: Front Pharmacol. 2018 Jun 29;9:687. doi: 10.3389/fphar.2018.00687 (PMC6036281; doi:10.3389/fphar.2018.00687)
Supplement: Supplementary file 1 [file Table_1.doc]

IGF2/IGF1R/Nanog signaling pathway regulates the proliferation of acute myeloid leukemia stem cells

Dan-dan Xu^1,2,3#^, Ying Wang^1#^, Peng-jun Zhou^1#^, Shu-rong Qin^1^, Rong Zhang^4^, Yi Zhang^5^, Xue Xue^3^, Jianping Wang^3^, Xia Wang^3^, Hong-Ce Chen^6^, Xiao Wang^1^, Yu-wei Pan^7^, Li Zhang^1^, Hai-zhao Yan^8^, Qiu-ying Liu^1^, Zhong Liu^1^, Su-hong Chen^1,3*^,Hong-yuan Chen^6*^, Yi-fei Wang^1,2*^

^#^These authors contributed equally to this work.

^*^Correspondence: Yi-fei Wang, Ph.D., College of Life Science and Technology, Jinan University, Guangzhou, 510632, P.R. China and Institute of Biomedicine, Jinan University, Guangzhou, 510632, P.R. China. Tel: 020-85220908; Fax: 020-85220504; E-mail: twang-yf@163.com

Hongyuan Chen, Department of Pathogen Biology and Immunology, School of Basic Course, Guangdong Pharmaceutical University, Guangzhou 510006, P.R. China. Email: hychen1208@ 126.com

**Supplementary Figure Legends**

Supplementary Fig. S1. Stemness factors expression level in KG-1a-LSCs. Relative expression level was calculated using the 2^-(ΔΔCt)^ method with GAPDH as the reference gene (n=3).


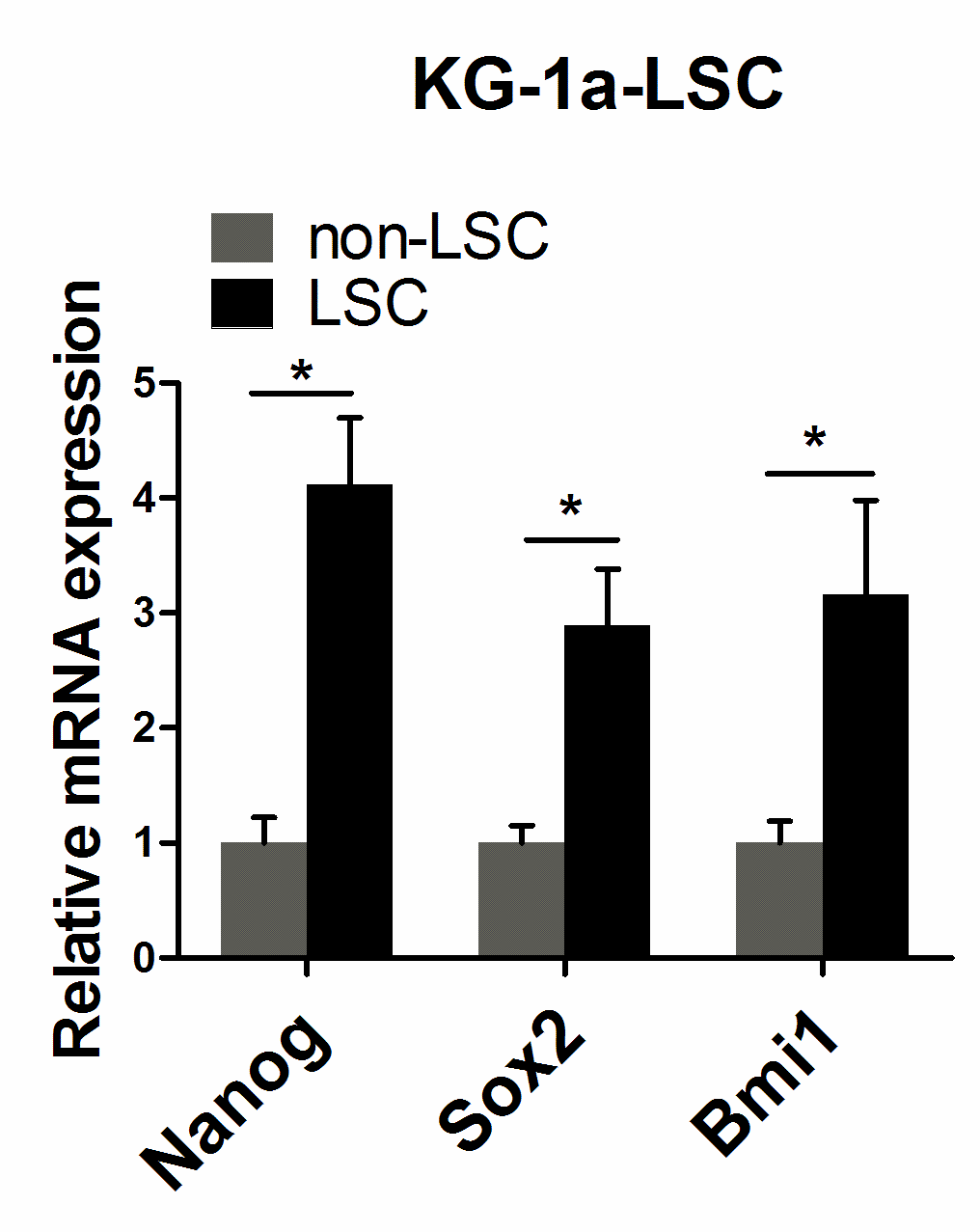


Supplementary Fig. S2. Representative images of colony efficiencies analysis of sphere formation after Nanog knockdown. Top agar contained single 1 × 10^4^ LSCs in IMDM medium with or without different concentration of IGF2, EGF, bFGF, B27. About two weeks later, the number of LSCs formation was counted and the colonies efficiency was calculated. Scale bar, 40 μm.


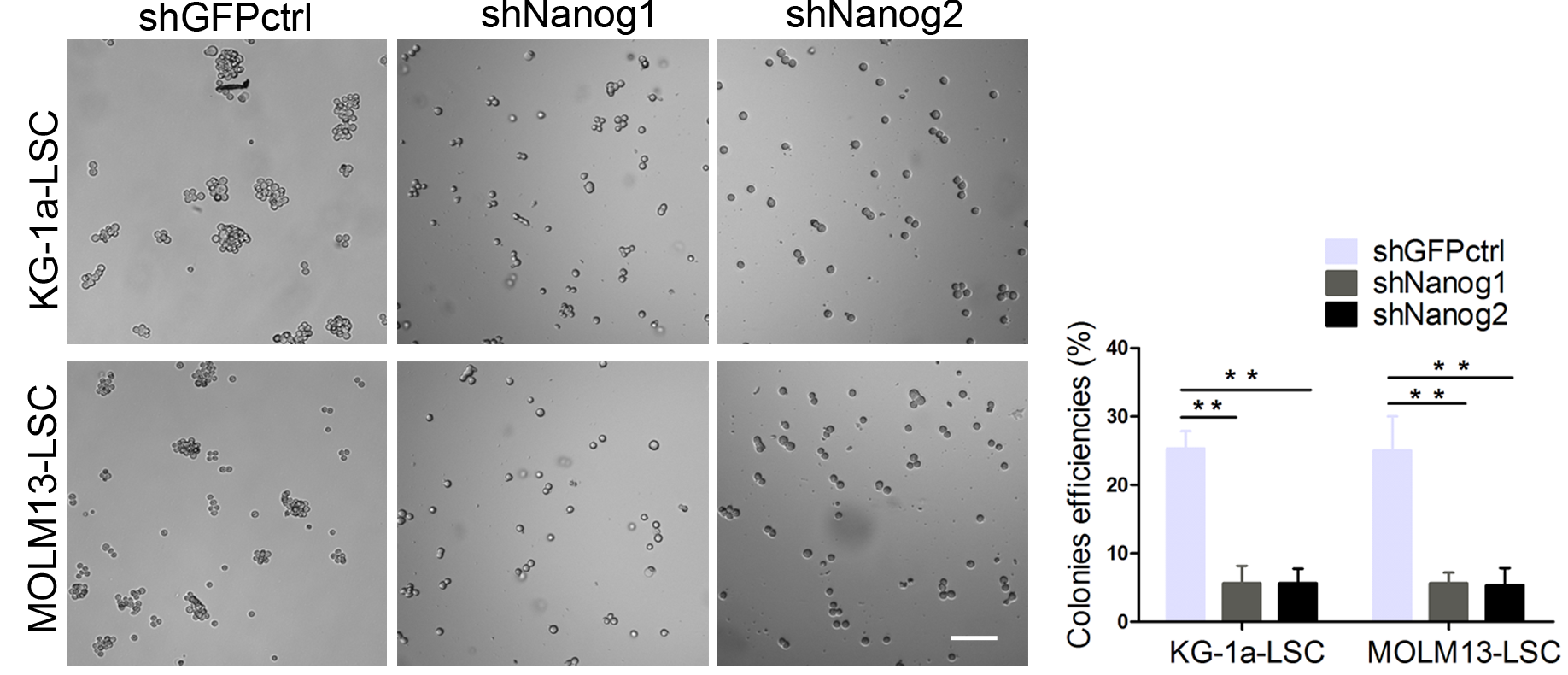


Supplementary Fig. S3 Nanog knockdown can abrogate the pro-proliferative effect of IGF2. For sphere formation assay, 1 × 10^4^ LSCs which were knockdown were cultured in 96-well plate with IMDM medium (STEMCELL Technologies, Vancouver, BC, Canada) with recombinant human EGF (20 ng/ml; Pepro Tech), recombinant human bFGF (20 ng/ml, Pepro Tech), B27 supplement (1:50, Invitrogen, Carlsbad, CA, USA) IGF2. Scale bar,100 μm.


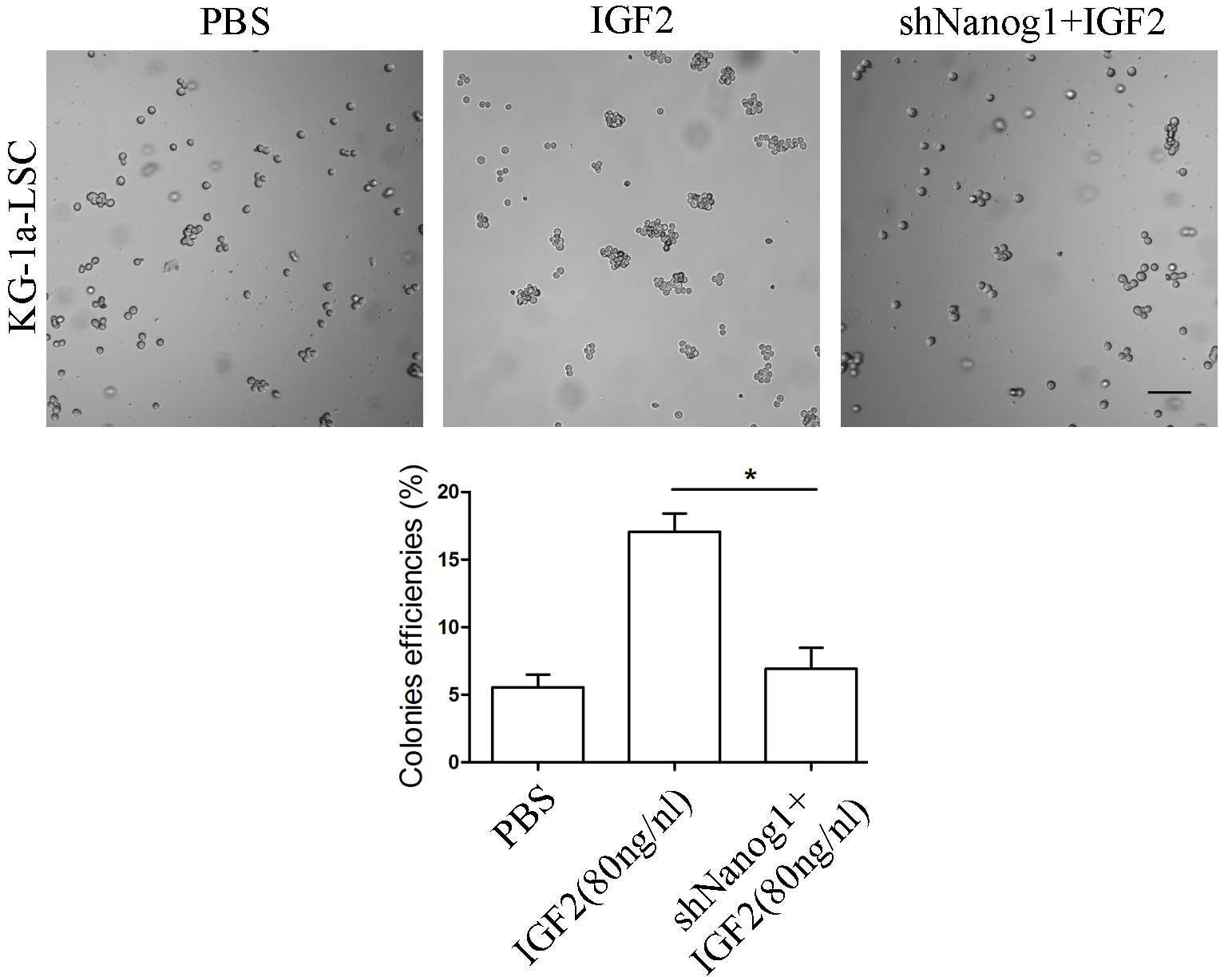


Supplementary Fig. S4 Soft agar plate assay demonstrate that Nanog is a downstream factor of IGF2 signaling pathway. PPP inhibited the LSCs proliferation. However, when Nanog was overexpressed it rescued the proliferation of LSCs partly. Nanog was overexprssed by pcDNA3.1-Nanog vector. Scale bar, 10mm.


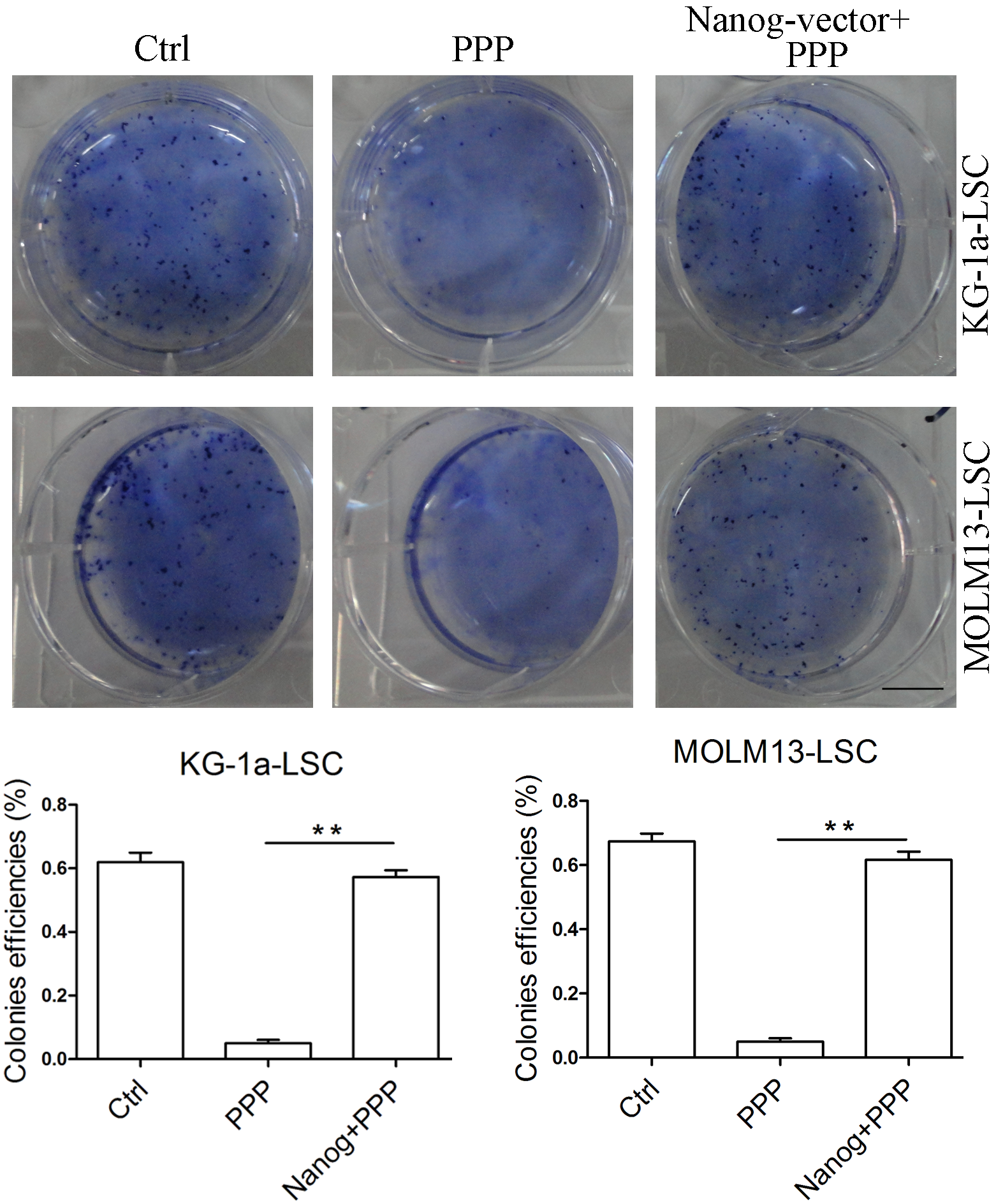


Supplementary Table S1. List of qPCR primers

| Gene name | Sequence(5’→3’) | |
| --- | --- | --- |
|  | Forward primer | Reverse primer |
| Nanog | ACCAGTCCCAAAGGCAAACA | GCTGGGTGGAAGAGAACACA |
| Sox2 | AGGAGAACCCCAAGATGCAC | TCTCCGTCTCCGACAAAAGT |
| Bmi1 | TGCAGCTCATCCTTCTGCTG | CCGATGCAATCTGTTCTGGTCA |
| IGF1R | GAAGGCGGGATGGAATGGAT | CCAGAGTATATCGCAATAACAGGA |

Supplementary Table S2. Summary of clinical characteristics of AML patients.

| No Age Sex PB/BM FAB WBC Platelet Hemoglobin  counts(10^9^/L) counts(10^9^/L) counts(g/L) | | | | | | | |
| --- | --- | --- | --- | --- | --- | --- | --- |
| 1 | 45 | male | PB | M1 | 15.4 | 56 | 84 |
| 2 | 52 | male | PB | M3 | 18.3 | 67 | 67 |
| 3 | 42 | female | PB | M1 | 17.2 | 94 | 53 |
| 4 | 70 | male | PB | M5 | 14.3 | 102 | 67 |
| 5 | 70 | female | PB | M5 | 13.4 | 78 | 72 |
| 6 | 56 | male | PB | M1 | 20.2 | 83 | 58 |
| 7 | 84 | male | PB | M5 | 18.7 | 59 | 46 |
| 8 | 42 | female | PB | M7 | 13.9 | 51 | 44 |
| 9 | 38 | male | PB | M3 | 16.4 | 109 | 59 |
| 10 | 44 | female | PB | M2 | 17.3 | 93 | 64 |
| 11 | 34 | male | BM | M5 | 13.2 | 73 | 35 |
| 12 | 53 | male | PB | M7 | 18.6 | 69 | 27 |
| 13 | 47 | female | PB | M3 | 19.5 | 82 | 69 |
| 14 | 62 | male | PB | M1 | 15.8 | 94 | 52 |
| 15 | 57 | male | PB | M5 | 13.2 | 71 | 47 |
| 16 | 44 | male | PB | M7 | 15.9 | 84 | 35 |

Abbreviations: FAB, French-American-Britain subtype; WBC, white blood cell; PB, peripheral blood; BM, bone marrow.
